# Supplementary material for: Regional connectivity drove bidirectional transmission of SARS-CoV-2 in the Middle East during travel restrictions
Source: Nat Commun. 2022 Aug 15;13:4784. doi: 10.1038/s41467-022-32536-1 (PMC9376901; doi:10.1038/s41467-022-32536-1)
Supplement: Supplementary file 3 — Reporting Summary [file 41467_2022_32536_MOESM3_ESM.pdf]

nature portfolio

Corresponding author(s): Edyth Parker

Last updated by author(s): Jul 21, 2022

Reporting Summary

Nature Portfolio wishes to improve the reproducibility of the work that we publish. This form provides structure for consistency and transparency in reporting. For further information on Nature Portfolio policies, see our Editorial Policies and the Editorial Policy Checklist.

Statistics

For all statistical analyses, confirm that the following items are present in the figure legend, table legend, main text, or Methods section.

n/a	Confirmed	
☐	☒	The exact sample size (*n*) for each experimental group/condition, given as a discrete number and unit of measurement
☐	☒	A statement on whether measurements were taken from distinct samples or whether the same sample was measured repeatedly
☐	☒	The statistical test(s) used AND whether they are one- or two-sided<br>*Only common tests should be described solely by name; describe more complex techniques in the Methods section.*
☐	☒	A description of all covariates tested
☐	☒	A description of any assumptions or corrections, such as tests of normality and adjustment for multiple comparisons
☐	☒	A full description of the statistical parameters including central tendency (e.g. means) or other basic estimates (e.g. regression coefficient) AND variation (e.g. standard deviation) or associated estimates of uncertainty (e.g. confidence intervals)
☐	☒	For null hypothesis testing, the test statistic (e.g. *F*, *t*, *r*) with confidence intervals, effect sizes, degrees of freedom and *P* value noted<br>*Give P values as exact values whenever suitable.*
☐	☒	For Bayesian analysis, information on the choice of priors and Markov chain Monte Carlo settings
☒	☐	For hierarchical and complex designs, identification of the appropriate level for tests and full reporting of outcomes
☒	☐	Estimates of effect sizes (e.g. Cohen's *d*, Pearson's *r*), indicating how they were calculated

Our web collection on statistics for biologists contains articles on many of the points above.

Software and code

Policy information about availability of computer code

Data collection	The intervention timeline from primary sources was validated against the timeline from the Oxford Covid-19 Government Response Tracker (OxCGRT)
Data analysis	Sequencing data was processed with iVar v1.2.2, bwa-mem v2.2, Snakemake 7.8.5, samtools 1.15.1. Sequences were aligned with Mafft v 7.505. Temporal regression performed with TreeTime 0.8.3. Pangolin lineages assigned with Pangolin nomenclature tool (version 3.1.11). Phylogenetic analyses were performed with IQtree2 v 1.6.12 and BEAST v 1.10 prerelease version 5. BEAST results processed with TreeAnnotator 1.10 and TreeMarkovJumpHistoryAnalyzer in BEAST v 1.10 prerelease version 5. Custom code was used to calculate the export and import indices -- freely available at the paper associated github (https://github.com/andersenlab/paper_2022_jordan-sars2-phylogenetics). The dynamic time warp analysis was performed using the DTAIDistance module for python v3. Epidemic analyses was performed with the Epinow2 package in R, with data accessed from outbreak.info with the outbreak.info package for R. FoldX v5 was used for structural modelling of Q957L. Bray--curtis distances calculated with vegan package in R. Trees were visualized using baltic (https://github.com/evogytis/baltic). Selection analyses performed with MEME (Mixed Effects Model of Evolution) model in Hyphy v2.5.27.

For manuscripts utilizing custom algorithms or software that are central to the research but not yet described in published literature, software must be made available to editors and reviewers. We strongly encourage code deposition in a community repository (e.g. GitHub). See the Nature Portfolio guidelines for submitting code & software for further information.

Data

Policy information about availability of data

All manuscripts must include a data availability statement. This statement should provide the following information, where applicable:

- Accession codes, unique identifiers, or web links for publicly available datasets
- A description of any restrictions on data availability
- For clinical datasets or third party data, please ensure that the statement adheres to our policy

The data for our analyses can be found at https://github.com/andersen-lab/paper_2022_jordan-sars2-phylogenetics. All sequence data generated were made available via the GISAID database (accessions available https://github.com/andersen-lab/HCoV-19-Genomics) and NCBI under BioProject ID PRJNA612578. . Travel data available on request.

Human research participants

Policy information about studies involving human research participants and Sex and Gender in Research.

Reporting on sex and gender	Information not collected or reported.
Population characteristics	Samples that were sequenced were collected from routine diagnostic testing, with no metadata available to authors.
Recruitment	We collected SARS-CoV2 samples from routine diagnostic tests performed by Biolab Diagnostic Laboratories, which has 20 branches in five governorates including major cities Amman, Irbid, and Zarqa. We collated samples from walk-in/drive-through testing centers, public and private referral laboratories, house-call services as well as health care facilities. Five to ten percent of total samples with a Ct value <32 in the TaqPath COVID-19 PCR assay were selected at random for sequencing across the major cities. Selection biases are introduced as 1) Asymptomatic and less severe cases are less likely to be sampled/sequenced if they do not undergo testing 2) Healthcare seeking/socioeconomic biases as testing is performed in the private sector. Less testing in asymptomatic/ more mild cases may result in biasing of lineage detection/dynamics to lineages that cause more severe disease.
Ethics oversight	The Institutional Review Boards (IRBs) at Scripps Research Institute (TSRI) (IRB-21-7739) and the Cell Therapy Center of The University of Jordan (IRD-CTC/1-2020/01). Statement added: "A consent waiver was requested and approved by IRB as this study uses pre-existing RNA obtained anonymously from various Biolab collection points, with no risk to subjects."

Note that full information on the approval of the study protocol must also be provided in the manuscript.

Field-specific reporting

Please select the one below that is the best fit for your research. If you are not sure, read the appropriate sections before making your selection.

☒ Life sciences ☐ Behavioural & social sciences ☐ Ecological, evolutionary & environmental sciences

For a reference copy of the document with all sections, see nature.com/documents/nr-reporting-summary-flat.pdf

Life sciences study design

All studies must disclose on these points even when the disclosure is negative.

Sample size	N=579 sequences were generated during the study. No formal statistical approach was used to determine sample size. Five to ten percent of total samples with a Ct value <32 in the TaqPath COVID-19 PCR assay were selected at random for sequencing across the major cities. The final sample size was governed by experimental and economic feasibility.
Data exclusions	None.
Replication	Estimates were assessed across three replicates of three downsampling strategies to account for global sequencing biases. Our findings were robust to randomization and downsampling strategies. We also compared estimates to non-sample dependent estimates from indices calculated from travel and incidence data, which also showed high agreement to our genomic estimates (formally tested).
Randomization	Samples were not randomized. Not applicable.
Blinding	Not applicable as patient data metadata was not known or used in analyses.

Reporting for specific materials, systems and methods

We require information from authors about some types of materials, experimental systems and methods used in many studies. Here, indicate whether each material, system or method listed is relevant to your study. If you are not sure if a list item applies to your research, read the appropriate section before selecting a response.

Materials & experimental systems

n/a	Involved in the study
☒	☐ Antibodies
☐	☒ Eukaryotic cell lines
☒	☐ Palaeontology and archaeology
☒	☐ Animals and other organisms
☒	☐ Clinical data
☒	☐ Dual use research of concern

Methods

n/a	Involved in the study
☒	☐ ChIP-seq
☐	☒ Flow cytometry
☒	☐ MRI-based neuroimaging

Eukaryotic cell lines

Policy information about cell lines and Sex and Gender in Research

Cell line source(s)	Both HEK293T and Calu-3 cell lines were obtained from ATCC
Authentication	None of the cell lines used were authenticated
Mycoplasma contamination	All cell lines are routinely tested for mycoplasma contamination using commercial PCR test kits.
Commonly misidentified lines (See ICLAC register)	N.A.

Flow Cytometry

Plots

Confirm that:

☒ The axis labels state the marker and fluorochrome used (e.g. CD4-FITC).

☒ The axis scales are clearly visible. Include numbers along axes only for bottom left plot of group (a 'group' is an analysis of identical markers).

☒ All plots are contour plots with outliers or pseudocolor plots.

☒ A numerical value for number of cells or percentage (with statistics) is provided.

Methodology

Sample preparation	Transduced Calu-3 cells were trypsinized and resuspended in PBS-2%FBS and analyzed by flow cytometry
Instrument	BD Celesta
Software	Data Collection: BD FACSDiva<br>Data Analysis: FlowJo
Cell population abundance	N.A. no sorting of populations involved in this study.
Gating strategy	Preliminary gating for live cells by SSC/FSC, and further secondary gating for single cells. Gating of GFP positive cells was performed by using non-transduced, GFP negative, Calu-3 cells.

☒ Tick this box to confirm that a figure exemplifying the gating strategy is provided in the Supplementary Information.
